# Burnout, employee engagement, and changing organizational contexts in VA primary care during the early COVID-19 pandemic

**DOI:** 10.1186/s12913-023-10270-8

**Published:** 2023-11-27

**Authors:** Eric A. Apaydin, Danielle E. Rose, Michael R. McClean, David C. Mohr, Elizabeth M. Yano, Paul G. Shekelle, Karin M. Nelson, Rong Guo, Caroline K. Yoo, Susan E. Stockdale

**Affiliations:** 1 Center for the Study of Healthcare Innovation, Implementation & Policy, VA Greater Los Angeles Healthcare System, 11301 Wilshire Blvd. (151), Los Angeles, CA 90073 USA; 2 RAND Corporation, Santa Monica, CA USA; 3 National Center for Organization Development, Veterans Health Administration, Cincinnati, OH USA; 4 Department of Health Law, Policy & Management, School of Public Health, Boston University, Boston, MA USA; 5 Department of Health Policy and Management, Fielding School of Public Health, University of California, Los Angeles, Los Angeles, CA USA; 6 Department of Medicine, David Geffen School of Medicine, University of California, Los Angeles, CA USA; 7 Seattle-Denver Center of Innovation, VA Puget Sound Health Care System, Seattle, WA USA; 8 Division of General Internal Medicine, Department of Medicine, University of Washington School of Medicine, University of Washington, Seattle, WA USA; 9 Department of Psychiatry and Biobehavioral Sciences, David Geffen School of Medicine, University of California, Los Angeles, Los Angeles, CA USA

**Keywords:** Burnout, Primary care, Healthcare workforce, Employee engagement, COVID-19, Virtual care

## Abstract

**Background:**

The COVID-19 pandemic involved a rapid change to the working conditions of all healthcare workers (HCW), including those in primary care. Organizational responses to the pandemic, including a shift to virtual care, changes in staffing, and reassignments to testing-related work, may have shifted more burden to these HCWs, increasing their burnout and turnover intent, despite their engagement to their organization. Our objectives were (1) to examine changes in burnout and intent to leave rates in VA primary care from 2017–2020 (before and during the pandemic), and (2) to analyze how individual protective factors and organizational context affected burnout and turnover intent among VA primary care HCWs during the early months of the pandemic.

**Methods:**

We analyzed individual- and healthcare system-level data from 19,894 primary care HCWs in 139 healthcare systems in 2020. We modeled potential relationships between individual-level burnout and turnover intent as outcomes, and individual-level employee engagement, perceptions of workload, leadership, and workgroups. At healthcare system-level, we assessed prior-year levels of burnout and turnover intent, COVID-19 burden (number of tests and deaths), and the extent of virtual care use as potential determinants. We conducted multivariable analyses using logistic regression with standard errors clustered by healthcare system controlled for individual-level demographics and healthcare system complexity.

**Results:**

In 2020, 37% of primary care HCWs reported burnout, and 31% reported turnover intent. Highly engaged employees were less burned out (OR = 0.57; 95% CI 0.52–0.63) and had lower turnover intent (OR = 0.62; 95% CI 0.57–0.68). Pre-pandemic healthcare system-level burnout was a major predictor of individual-level pandemic burnout (*p* = 0.014). Perceptions of reasonable workload, trustworthy leadership, and strong workgroups were also related to lower burnout and turnover intent (*p* < 0.05 for all). COVID-19 burden, virtual care use, and prior year turnover were not associated with either outcome.

**Conclusions:**

Employee engagement was associated with a lower likelihood of primary care HCW burnout and turnover intent during the pandemic, suggesting it may have a protective effect during stressful times. COVID-19 burden and virtual care use were not related to either outcome. Future research should focus on understanding the relationship between engagement and burnout and improving well-being in primary care.

## Introduction

Crises such as the COVID-19 pandemic can have widespread impacts on the healthcare system, resulting in organizational changes that can negatively impact healthcare worker burnout and turnover. During previous crises, like the 2005 Kashmir earthquake [1], the 2011 Fukushima earthquake, tsunami, and nuclear incident [2], and 2017 Harvey and Maria hurricanes [3], burnout and other mental health issues increased among healthcare, disaster, and related workers. Healthcare worker (HCW; i.e., providers and staff) burnout, primarily characterized by emotional exhaustion and depersonalization, [4] has been widespread across many specialties, in the US [5, 6] and globally [7–9], during the COVID-19 pandemic. Turnover intent, or the intent to leave one’s job, among HCWs was also high in the US [10] and internationally [11, 12] during the pandemic. Prior to the pandemic, the burnout phenomenon [13] was associated with both individual and organizational drivers [14], and linked to numerous negative healthcare consequences [14–16], including increased medical errors, reduced patient satisfaction, poorer quality of care, and increased turnover**.** Similarly, previous research has identified turnover as a possible driver of primary care shortages, [17, 18] and found that it is very expensive, with the estimated costs of replacing a single HCW ranging from $14,000 for a medical assistant [19] to $1,000,000 for a physician [20].

The COVID-19 pandemic tested the capacity of healthcare systems to respond to a crisis, requiring healthcare system administrators to adapt organizational structures and processes overnight and changing organizational contexts in ways that may have contributed to higher levels of burnout and turnover among primary care healthcare workers (HCWs). Little is known, however, about how COVID crisis-induced changes may have impacted primary care HCW burnout. Crisis levels of acute and emergent care often required surge staffing, [21] which may have included temporarily reassigning primary care HCWs into COVID-related screening or inpatient care. These staffing changes may have contributed to increased burnout among the reassigned HCWs exposed to new stressors and among the remaining HCWs with increased workloads. Those HCWs with higher workloads may have experienced increased burnout, as has been documented in research prior to the pandemic [14]. In addition, the rapid shift towards virtual care (e.g., video- or telephone-based care), [22] due to COVID-related quarantining and social distancing restrictions, may have increased primary care HCW burnout via changes in work flows, workload, learning how to use the virtual care platforms, and/or educating patients on how to use the modality [10, 23]. Conversely, virtual care use may have reduced HCW stress and associated burnout by reducing the risk of COVID-19 infection.

Some characteristics of individual HCWs have been shown to be protective against burnout and turnover. Specifically, more engaged employees may be more resilient and less susceptible to burnout. Engagement is characterized by energy, involvement, and effectiveness on the job [24]. A systematic review and meta-analysis [25] of burnout and engagement in 37 studies across job fields showed that higher engagement and lower burnout were consistently correlated with fewer health complaints, more job satisfaction, and more organizational commitment. To date, employee engagement has not been well-studied in primary care. Protective factors, like employee engagement and positive working environments (e.g., a workplace with a sense of community), may attenuate increases in HCW burnout [5] and turnover intentions [26], but it is unknown whether engagement can buffer the impact of crisis-induced organizational changes on burnout and turnover.

Good leadership and strong workgroups may have also been protective of burnout among individual HCWs during the pandemic. High quality leadership, characterized by values like trust, respect, mentorship, and inspiration, has been consistently associated [14, 27] with lower burnout among physicians and other HCWs. Coworkers also matter – evidence suggests that in VA primary care, workgroups with good collaboration and communication and high competency [28–30] have members that are less likely to be burned out. Strong leadership and good relationships with coworkers were likely very important during the chaotic times of the early pandemic, and these characteristics may have protected against higher HCW burnout.

Our objectives in this study were to 1) examine changes in reported VA primary care HCW burnout and turnover intent prior to and during the early COVID-19 crisis and 2) examine how individual protective characteristics (engagement, and perceptions of workload, leadership, and workgroups) and COVID-related organizational contextual factors (via COVID-19 burden, virtual care use, and prior year burnout and turnover intent), were associated with burnout and turnover intent during the first six months of the pandemic.

## Methods

### Data sources

Data sources included survey data from the 2017 to 2020 VA All Employee Surveys (AES) and 2020 administrative data from the VA COVID Shared Data Resource (COVID SDR), the VA Corporate Data Warehouse (CDW), and the VHA Service Support Center (VSSC).

The AES is a yearly survey of VA employee attitudes [31] and is typically administered in June (see Table 1 for yearly response rates), except in 2020 when COVID delayed administration until September. The AES is anonymous at the individual-level, with identifiers for an individual’s healthcare system. Each annual survey is cross-sectional, and responses are not linked across years. The CDW is a national repository of VA clinical and administrative information that includes clinical, administrative, financial, enrollment, and benefits data [32]. The VSSC contains information on healthcare system complexity, detailed below. The COVID SDR [33] was specifically created at the beginning of the pandemic by the VA to pool COVID-19 clinical data from all VA healthcare systems.

**Table 1:** Average burnout and turnover intent for VA primary care HCWs from 2017–20 AES

Year	Response rate (VA-wide)	Burnout (% EE or DP > = once a week or more)	n/d	Turnover Intent (%, 1 year)	n/d
2017	59.5	41.6	6170/14825	33.0	4821/14623
2018	61.6	35.7	5781/16201	34.5	5720/16649
2019	63.9	35.4	6191/17504	34.3	6177/18017
2020	69.4	37.2	7320/19680	30.6	6096/19894

*Abbreviations*: *AES* All Employee Survey, *d* denominator who answered question, *DP* depersonalization, *EE* emotional exhaustion, *n* numerator who responded to question affirmatively, *VA* Veterans Health Administration

### Sample

The sample included primary care HCWs who completed the AES surveys from 2017–2020. Data from 2017–19 was also used to describe pre-pandemic trends and to create healthcare system-level predictors for burnout and turnover intent for the year prior to the beginning of the pandemic (2019). Eligible respondents indicated that they worked on a VA primary care team in one of the following professions: primary care provider (PCP: physician [MD/DO], nurse practitioner [NP], and physician assistant [PA]), registered nurse (RN), clinical associate (licensed practical nurse [LPN], licensed vocational nurse [LVN], nursing assistant, intermediate care technician, and health technician), and administrative associate (administrative or clerical employee working in a clinical area, or a general administrative employee).

### Measures

#### Outcomes

The outcomes included individual-level burnout and turnover intent. Burnout was assessed using a single-item measure for emotional exhaustion (“I feel burned out from my work.”) and a single-item measure for depersonalization (“I worry that this job is hardening me emotionally.”). Both were measured on a 7-point frequency scale (never, a few times a year or less, once a month or less, a few times a month, once a week, a few times a week, or every day). For analysis, we created a dichotomous measure of burnout, with 1 = experiencing symptoms once a week or more versus 0 = less than once per week on either the emotional exhaustion or the depersonalization item, as in previous studies [34, 35]. Similarly, turnover intent was a dichotomous measure, with 1 = a “yes” response (“yes, but taking another job within VA”, “yes, to retire,” “yes, to take another job within the federal government,” “yes, to take another job outside the federal government,” and “yes, other”) to the question of “Are you considering leaving your job in the next year, and if so why?” and 0 = “no”.

#### Individual-level protective factors

We also include several individual-level factors that that may be protective against burnout and turnover intent.

The first, employee engagement, was a factor that we hypothesize may be protective against crisis-induced organizational stressors, and that previous studies have suggested might be protective against burnout and turnover intent. Individual-level employee engagement was assessed using a 5-point agreement scale ranging from “strongly disagree” to “strongly agree,” ranked from 1 to 5, for four statements: “I recommend my organization as a good place to work.”; “This organization really inspires the very best in me in the way of job performance.”; “I always do more than is actually required.”; and “My job is more than just a paycheck to me.” Respondents were rated as highly engaged if they agreed or strongly agreed with most items in this set (i.e., a score of 18 or above) [36]. All four items loaded onto one factor with an eigenvalue greater than 1 (Cronbach’s alpha = 0.76).

We also included three other individual-level factors that may be associated with burnout and turnover intent: perceptions of workload, leadership, and workgroup. These individual factors and their corresponding survey items are as follows: reasonable workload (“My workload is reasonable.”), supervisor listening (“My supervisor listens to what I have to say.”), supervisor respect (“My supervisor treats me with respect.”), supervisor trust (“I have trust and confidence in my supervisor.”), workgroup cooperation (“The people I work with cooperate to get the job done.”), workgroup competency (“My work unit has the job-relevant knowledge and skills necessary to accomplish organizational goals.”), and workgroup collaboration (“Workgroups collaborate to accomplish shared objectives.”). All of these factors were assessed using 5-point agreement scales, ranging from “strongly disagree” to “strongly agree,” and ranked from 1 to 5. Respondents who “agreed” or “strongly agreed” with a factor’s survey item were considered to endorse that factor.

#### Healthcare system-level predictors of burnout and turnover intent

Organizational contextual factors specific to the COVID crisis included healthcare system-level COVID-19 burden, virtual care use, and prior year average HCW burnout and turnover intent. Using data from the COVID SDR, COVID-19 burden was operationalized as the number of COVID-19 tests administered and deaths per 1000 patients from 03/15/20 to 09/15/20 (i.e., the first six months of the pandemic and six months prior to the administration of the AES 2020 survey). These measures were averaged by healthcare system and grouped into quartiles (tests) or terciles (deaths) for analysis, to adjust for uneven distribution of data and ease interpretation of results. Testing rates were grouped into four categories: 9.2 to 38.7 (lowest quartile), 38.9 to 47.6 (2^nd^ quartile), 47.8 to 61.4 (3^rd^ quartile), and 65.2 to 471.7 tests per 1000 unique patients (highest quartile). Death rates were grouped into three categories: 0.0 to 0.27 (lowest tercile), 0.27 to 0.69 (middle tercile), and 0.69 to 3.65 deaths per 1000 unique patients (highest tercile).

The number of primary care visits delivered virtually by patient location (including video, phone and supplementary remote [e.g., store-and-forward video, audio, or image messages, or specialty visits like tele-smoking cessation]) [22] and in-person from 03/15/20 to 09/15/20 by healthcare system were extracted from the CDW. A measure for healthcare system-level proportion of virtual care use was created by dividing total virtual primary care visits by all primary care visits (in-person + virtual visits) for each healthcare system.

We used 2019 AES data to create a measure of the proportion of primary care HCWs with high emotional exhaustion or depersonalization in 2019 by healthcare system, using the same definition of dichotomous burnout described above. A similar healthcare system-level turnover intent predictor variable was also constructed using 2019 AES data.

#### Other covariates

Individual-level demographic covariates included professional role (provider, registered nurse, clinical associate, or administrative associate); gender (male or female); race (white, black/African American, Asian, American Indian or Alaskan Native, or Native Hawaiian or other Pacific Islander); ethnicity (non-Hispanic or Hispanic); age (29 and under, 30–49, or 50 +); VA tenure (less than 2 years, between 2 and 10 years, between 10 and 20 years, or more than 20 years); and supervisor status (yes or no).

We also included a healthcare system-level complexity measure using the VHA Complexity Model [37] categories available from VSSC: group 1 (combining groups 1a, 1b, and 1c; most complex), group 2, and group 3 (least complex). In general, group 1 healthcare systems have medium-to-high patient volumes, medium-to-high risk patients, more complex clinical programs, and have medium-to-large teaching and research programs [38]. Group 2 and 3 healthcare systems generally have low-to-medium patient volumes, lower risk patients, and less complex clinical programs. Both group 2 nor 3 healthcare systems typically have small or no teaching or research programs.

### Statistical analyses

We used descriptive statistics to characterize study outcomes, predictors, and covariates. To compare burnout and turnover intent levels before and during the pandemic crisis period, we examined the prevalence of burnout and turnover intent among VA primary care HCWs from the 2017 to 2020 administrations of the AES.

For multivariable analyses, individual HCW survey responses for 2020 were linked to 2020 and 2019 healthcare system-level (e.g., VA hospital-based medical center and its affiliated clinics) data. We used logistic regression, modeling burnout and turnover separately, and including employee engagement; perceptions of workload, leadership, and workgroup; COVID-19 burden; virtual care use; and prior year burnout as predictors. Models also included demographics and healthcare system complexity as control variables, with cluster adjustments at the healthcare system level.

## Results

### Historical trends in VA primary care burnout and turnover intent

VA primary care HCWs reported burnout rates of 35–42% from 2017–2020 (Table 1), with rates declining from 42% in 2017 to 35–36% in 2018–2019, and then increasingly slightly to 37% during the early COVID-19 pandemic. These HCWs also reported one-year turnover intention rates of 31–34% from 2017–2020, with a decrease in turnover intent during the early pandemic (2020). Response rates to the AES among all employees during this period ranged from 60–69%.

### Descriptive statistics for 2020 analytic sample

In 2020, high emotional exhaustion and depersonalization burnout were reported by 34.2% and 26.6% of respondents, respectively (Table 2). In the same year, almost 31% of the sample indicated their intent to leave VA within the next year. Nearly 40% of respondents reported high employee engagement, and over half (57.4%) stated that their workload was reasonable. Over 70% of respondents felt that their supervisors listened, were respectful and were trustworthy, and over 60% agreed that their workgroups cooperated, collaborated, and were competent.

**Table 2:** Individual characteristics (*n* = 19,894)

Characteristic	n	%
*Professional role*		
Provider (MD/DO, NP, PA)	4,851	24.4
RN	6,635	33.4
Clinical associate	5,877	29.5
Administrative associate	2,531	12.7
*Gender*		
Male	4,544	22.8
Female	14,824	74.5
Unknown	526	2.6
*Race*		
White	11,241	56.5
Black or African American	4,199	21.1
Asian	2,216	11.1
American Indian or Alaskan Native	490	2.5
Native Hawaiian or other Pacific Island	381	1.9
Unknown	1,367	6.9
*Ethnicity*		
Non-Hispanic	15,680	78.8
Hispanic	1,796	9.0
Unknown	2,418	12.2
*Age*		
29 and under	684	3.4
30–49	9,028	45.4
50 +	9,534	47.9
Unknown	648	3.3
*VA tenure*		
Less than 2 years	4,494	22.6
Between 2 and 10 years	8,841	44.4
Between 10 and 20 years	4,477	22.5
More than 20 years	1,624	8.2
Unknown	458	2.3
*Supervisor status*		
No	14,143	71.1
Yes	5,445	27.4
Unknown	306	1.5
*High employee engagement (mostly agree or strongly agree on four items)*		
No	12,005	60.3
Yes	7,889	39.7
*High EE or DP burnout (once a week or more for either)*		
No	12,360	62.1
Yes	7,320	36.8
Unknown	214	1.1
*High EE burnout (once a week or more)*		
No	12,801	64.4
Yes	6,798	34.2
Unknown	295	1.5
*High DP burnout (once a week or more)*		
No	14,288	71.8
Yes	5,285	26.6
Unknown	321	1.6
*Turnover intent in next year*		
No	13,798	69.4
Yes	6,096	30.6
*Reasonable workload (agree or strongly agree)*		
No	8,467	42.6
Yes	11,427	57.4
*Supervisor listening (agree or strongly agree)*		
No	5,043	25.4
Yes	14,851	74.7
*Supervisor respect (agree or strongly agree)*		
No	3,818	19.2
Yes	16,076	80.8
*Supervisor trust (agree or strongly agree)*		
No	5,789	29.1
Yes	14,105	70.9
*Workgroup cooperation (agree or strongly agree)*		
No	5,443	27.4
Yes	14,451	72.6
*Workgroup competency (agree or strongly agree)*		
No	4,485	22.5
Yes	15,409	77.5
*Workgroup collaboration (agree or strongly agree)*		
No	7,642	38.4
Yes	12,252	61.6

All data from 2020

*Abbreviations*: *DO* Doctor of Osteopathy, *DP* depersonalization, *EE* emotional exhaustion, *MD* Doctor of Medicine, *RN* registered nurse, *VA* Veterans Health Administration

Respondents were mostly female (74.5%), white (56.5%), and non-Hispanic (78.8%). Most of the sample was 30 to 49 years of age (45.4%) or 50 years or older (47.9%), and nearly half had 2–10 years of VA experience (44.4%). Supervisors were the minority among respondents (27.4%), and registered nurses (33.2%) and clinical associates (29.3%) were the most common professions in the sample.

Organizational context regarding COVID burden (Table 3) showed high variation across healthcare systems for COVID-19 testing per 1000 patients (mean [M] 56.5, standard deviation [SD] 46.9; range [R] 9.2–471.7), COVID-specific mortality rates per 1000 patients (M 0.46; SD 0.52; R 0.0–3.65), and virtual primary care visits (M 29%, SD 21%, R 0–76%). Less variation was observed in prior year healthcare system-level average burnout (M 31%, SD 3%, R 24–41%) and intent to leave within one year (M 35%, SD 3%, R 26–44%).

**Table 3:** Healthcare system characteristics (*n* = 139)

**Characteristic**	**M**	**SD**	**Range**
Healthcare system PC visits conducted virtually	29%	21%	0–76%
2020 healthcare system-level high burnout (EE or DP)	31%	3%	24–40%
2020 healthcare system-level turnover intent	31%	3%	22–40%
2019 healthcare system-level high burnout (EE or DP)	31%	3%	24–41%
2019 healthcare system-level turnover intent	35%	3%	26–44%
Healthcare system COVID-19 deaths per 1000 unique patients	0.46	0.52	0.0–3.65
Healthcare system COVID-19 tests per 1000 unique patients	56.1	46.9	9.2–471.7
	**N**	**%**	
*Healthcare system COVID-19 death rate terciles*			
Lowest tercile (0–0.27 deaths per 1000 unique patients)	59	42.6	
Middle tercile (0.27–0.69 deaths per 1000 unique patients)	58	41.7	
Highest tercile (0.69–3.65 deaths per 1000 unique patients)	22	15.8	
*Healthcare system COVID-19 test quartiles*			
Lowest quartile (9.2–38.7 tests per 1000 unique patients)	47	33.8	
2^nd^ quartile (38.9–47.6 tests per 1000 unique patients)	30	21.6	
3^rd^ quartile (47.8–61.4 tests per 1000 unique patients)	27	19.4	
Highest quartile (65.2–471.7 tests per 1000 unique patients)	35	25.2	
*Healthcare system complexity*			
1 (most complex)	92	61.2	
2	20	14.4	
3 (least complex)	27	19.4	

All data from 2020, except 2019 burnout and turnover intent data

*Abbreviations*: *DP* depersonalization, *EE* emotional exhaustion, *M* mean, *PC* primary care, *SD* standard deviation

### Multivariable model results

In multivariable models (*n* = 16,191 [burnout model] and *n* = 16,333 [intent to leave model] in 139 healthcare systems), highly engaged employees were significantly less likely to report burnout (odds ratio [OR] 0.57; 95% confidence interval [CI] 0.52–0.63) or an intention to leave their jobs (OR 0.62; 95% CI 0.57–0.68; Table 4), adjusting for professional role, gender, age, race, ethnicity, VA tenure, and supervisor status.

**Table 4:** Odds of burnout and turnover intent by healthcare system and individual characteristics

	**EE or DP burnout**		**Turnover intent**	
	***n* = 16,191 in 139 healthcare systems**		***n* = 16,333 in 139 healthcare systems**	
**Characteristic**	**OR**	**95% CI**	**OR**	**95% CI**
*High employee engagement*				
No	Ref		Ref	
Yes	0.57*	0.52–0.63	0.62*	0.57–0.68
*Reasonable workload*				
No	Ref		Ref	
Yes	0.26*	0.24–0.28	0.54*	0.50–0.58
*Supervisor listening*				
No	Ref		Ref	
Yes	0.91	0.78–1.06	0.83*	0.73–0.95
*Supervisor respect*				
No	Ref		Ref	
Yes	1.04	0.89–1.21	0.85*	0.74–0.98
*Supervisor trust*				
No	Ref		Ref	
Yes	0.71*	0.62–0.82	0.69*	0.62–0.78
*Workgroup cooperation*				
No	Ref		Ref	
Yes	0.78*	0.72–0.85	0.77*	0.69–0.85
*Workgroup competency*				
No	Ref		Ref	
Yes	0.82*	0.74–0.91	0.78*	0.69–0.87
*Workgroup collaboration*				
No	Ref		Ref	
Yes	0.74*	0.67–0.80	0.77*	0.70–0.85
2019 healthcare system-level high burnout (EE or DP)	6.75*	1.47–30.94	–	–
2019 healthcare system-level high turnover intent	–	–	2.93	0.59–14.50
*Healthcare system COVID-19 death terciles*				
Lowest third (0–0.27 deaths per 1000 unique patients)	Ref		Ref	
Middle third (0.27–0.69 deaths per 1000 unique patients)	0.99	0.91–1.07	0.92	0.83–1.02
Highest third (0.69–3.65 deaths per 1000 unique patients)	0.91	0.81–1.03	0.84*	0.72–0.98
*Healthcare system COVID-19 test quartiles*				
Lowest quartile (9.2–38.7 tests per 1000 unique patients)	Ref		Ref	
2nd quartile (38.9–47.6 tests per 1000 unique patients)	0.99	0.90–1.10	1.03	0.90–1.18
3rd quartile (47.8–61.4 tests per 1000 unique patients)	0.92	0.82–1.02	0.97	0.84–1.13
Highest quartile (65.2–471.7 tests per 1000 unique patients)	0.91	0.79–1.05	0.98	0.86–1.13
Healthcare system proportion of virtual PC visits per all visits	1.00	0.83–1.22	0.86	0.70–1.05
*Healthcare system complexity*				
1	Ref		Ref	
2	0.99	0.85–1.14	0.92	0.76–1.11
3	1.01	0.89–1.16	0.97	0.83–1.12
*Professional role*				
Provider (MD/DO, NP, PA)	Ref		Ref	
RN	0.72*	0.64–0.79	1.27*	1.14–1.41
Clinical associate	0.71*	0.63–0.80	1.12	1.00–1.25
Administrative associate	0.87*	0.76–0.99	2.23*	1.93–2.57
*Gender*				
Male	Ref		Ref	
Female	1.00	0.92–1.08	0.75*	0.69–0.81
*Race*				
White	Ref		Ref	
Black or African American	0.80*	0.73–0.88	1.11*	1.01–1.22
Asian	0.87*	0.76–0.99	0.80*	0.69–0.93
American Indian or Alaskan Native	0.87	0.68–1.11	0.90	0.70–1.15
Native Hawaiian or other Pacific Island	1.13	0.88–1.46	1.44*	1.12–1.86
*Ethnicity*				
Non-Hispanic	Ref		Ref	
Hispanic	1.10	0.94–1.29	1.04	0.91–1.18
*Age*				
29 and under	Ref		Ref	
30–49	0.72*	0.60–0.86	0.80*	0.67–0.96
50 +	0.52*	0.43–0.63	0.74*	0.61–0.89
*VA tenure*				
Less than 2 years	Ref		Ref	
Between 2 and 10 years	1.60*	1.46–1.77	1.29*	1.16–1.44
Between 10 and 20 years	1.77*	1.57–1.99	1.30*	1.14–1.47
More than 20 years	1.81*	1.52–2.15	1.68*	1.44–1.97
*Supervisor status*				
No	Ref			
Yes	1.18*	1.08–1.29	1.12*	1.02–1.22

^*^ = *p* < 0.05; All data from 2020, except 2019 burnout and turnover intent data

*Abbreviations*: *CI* confidence interval, *DO* Doctor of Osteopathy, *DP* depersonalization, *EE* emotional exhaustion, *MD* Doctor of Medicine, *OR* odds ratios, *Ref* reference category, *RN* registered nurse, *VA* Veterans Health Administration

Reasonable workload was also associated with lower burnout (OR 0.26, 95% CI 0.24–0.28) and lower turnover intent (OR 0.54, 95% CI 0.50–0.58). Supervisor trust (burnout model: OR 0.71, 95% CI 0.62–0.82; turnover intent model: OR 0.69, 95% CI 0.62–0.78), and workgroup cooperation (burnout model: OR 0.78, 95% CI 0.72–0.85; turnover intent model: OR 0.77, 95% CI 0.69–0.85), competency (burnout model: OR 0.82, 95% CI 0.74–0.91; turnover intent model: OR 0.78, 95% CI 0.69–0.87), and collaboration (burnout model: OR 0.74, 95% CI 0.67–0.80; turnover intent model: OR 0.77, 95% CI 0.70–0.85) were also associated with lower likelihoods of both outcomes. Supervisor listening (turnover intent model: OR 0.83, 95% CI 0.73–0.95), and respect (turnover intent model: OR 0.85, 95% CI 0.74–0.98) were related to lower odds of turnover intent but not burnout.

Healthcare system-level COVID-19 tests and deaths, proportion of virtual care visits, and healthcare system complexity were not associated with either burnout or turnover intent. Average prior-year healthcare system-level burnout was significantly associated with 2020 individual-level burnout (OR 6.75; 95% CI 1.47–30.94) but not turnover intent. Prior year average healthcare system-level turnover intent was also not associated with current year individual-level turnover intent.

## Discussion

Trends in VA primary care burnout between 2017–2020 varied but hovered around 37%, with a high rate of 42% in 2017, suggesting little impact due to the early COVID-19 pandemic. Thirty-seven percent of VA primary care HCWs reported high burnout and nearly one-third reported turnover intent in September 2020. The lack of large increases in rates of burnout and turnover intent during 2020 may indicate the presence of local supports (e.g., strong leadership or good crisis response) that offset HCW burnout, or persistent non-COVID drivers of both outcomes in VA primary care.

Thirty-seven percent of VA primary care HCWs reported high burnout in 2020, and nearly 31% reported an intent to leave their job. Burnout rates among physicians and other advanced practitioners outside of VA ranged from 38 to 57% in late 2020 according to two national surveys, [39, 40] indicating that VA experienced less burnout during the early pandemic than community physicians. Turnover intent rates in late 2020 among non-VA physicians and advanced practitioners [39] were similar to the rates we found in among VA HCWs in our analysis. National data for nurses has not been published, but a state-level survey from New Jersey indicated that 37% of nurses intended to leave their jobs in late 2020, [41] which is higher in our sample of VA primary care HCWs (31%). In another state-wide analysis in early 2022, intent to leave was even higher among nurses in Michigan at 39% [42]. These comparator studies contain non-primary care specialties, so this analysis may underestimate the gap in burnout between VA and non-VA primary care HCWs, as burnout in both community and VA primary care is generally higher than in most other specialties [43, 44]. VA HCWs in primary care may have had even lower rates of burnout and turnover intent than equivalent HCWs in the community than what is suggested here.

We found that highly engaged HCWs were less likely to report either high burnout or their intent to leave practice in 2020. Engagement, commonly conceived as either the opposite of burnout [45] or an independent positive construct, [46] is a cultivated, rather than emergent, phenomenon [47, 48]. The Job Demands-Resources Model formalizes these differences between burnout and engagement, and proposes that burnout is driven by job demands (e.g., workload), whereas engagement is driven by job resources (e.g., high quality supervisors or leaders, social support from colleagues, etc.) [49]. Cultivating these resources in “normal” times can increase engagement, and potentially protect against a large increase in burnout that results from increases in demands during crises. Engagement has been previously associated with positive organizational culture in a small sample of nurses [50] (as conceived by the six areas of worklife: workload, control, reward, community, fairness, and values [26]) and with lower burnout across professions and countries [5, 26]. In our sample, high engagement was linked to lower burnout during the pandemic, suggesting that engagement may be a bulwark against drivers of burnout external to an organization. Our analyses also showed that healthcare system-level average burnout in 2019 was associated with individual-level burnout and turnover intent in 2020, suggesting the persistent and longitudinal relationship between an organizational culture of burnout and these individual outcomes.

Individual perceptions of reasonable workload, high quality leadership, and good workgroups were linked with lower rates of burnout and turnover intent in our data as well. Higher workload during the COVID-19 pandemic was found to be related to more burnout in a wide range of studies of HCWs conducted across the world [51]. Workloads pushing HCWs beyond their training or interfering with their personal lives [52] and a desire to decrease one’s workload, [7] were found to be particularly related to high burnout. Leadership, overall, [53] and in terms of communication and support [54], has also been previously linked to lower burnout among HCWs during the pandemic. The relationships between good workgroups, or teams, and burnout during the pandemic have not been extensively studied in the literature, but there is some evidence that team identification [55] and support [56] were related to lower pandemic-era burnout in primary care and emergency medicine. Finally, it is important to note that only workload had a stronger relationship with burnout than engagement in our findings, suggesting that engagement may encompass aspects of the working environment that are more than just the sum of workload, leadership, and workgroups. Perceptions of good leadership and workgroups were less related to decreased burnout, implying that these constructs were less protective than overall engagement among primary care HCWs during the early pandemic.

Our results also show that healthcare system-level COVID-19 burden, shift to virtual care use, and complexity were not associated with either high burnout or turnover intent. While high burnout has been reported among HCWs across specialties and geographies during the pandemic, [5–9] the relationships between primary care burnout or turnover intent and COVID-19 burden has not been widely studied. We found only one study conducted in a Belgian intensive care unit with nurses that found little association between burnout and the proportions of COVID-19 patients or deaths (to total patients or deaths) [57]. Similarly, the effect of virtual care on healthcare outcomes during the pandemic has been widely studied, [58] but its relationship with burnout or turnover intent has only been the subject of a few analyses. These previous studies suggest that greater telehealth self-efficacy (i.e., comfort with the use of telehealth or virtual care) [10] or fewer “difficulties with new technologies” [23] during the pandemic may be associated with lower burnout or turnover intent. Our results suggest that the COVID-19 crisis and associated virtual care use had no relationship with burnout or turnover intent. It is possible that neither COVID-19 nor virtual care use were as impactful as longer-term organizational contextual factors associated with burnout and turnover, such as staffing shortages and instability [29, 59].

Our study had a few notable limitations, including the inability to identify individuals in the anonymous AES data and match those data with prior-year individual-level AES data. In addition, our COVID-19 deaths and tests and virtual care data were healthcare system averages across the first six months of the pandemic (03/15/20 to 09/15/20) and may not have adequately captured variations due to local virus surges. Finally, our measures of COVID burden may not have reflected the true impact of the pandemic on primary care, including the uncertainties associated with staff reassignments, patient screening procedures, care management for vulnerable patients, and the sudden shift to virtual modalities.

More research is needed to explore whether interventions to improve job resources and employee engagement can protect HCW from the negative impacts of crises affecting healthcare delivery and other system-level changes that may increase stress among HCW. There is some evidence that evidence-based quality improvement, an implementation strategy for clinical guidelines and care models, may have reduced VA PCP burnout during patient-centered medical home implementation [60]. This reduction in burnout may have been related to engaging primary care HCWs in participatory decision-making and the empowerment of frontline HCWs to address workplace quality issues. In another study of PCPs and staff outside the VA, participants who did not help design burnout reduction interventions actually experienced an increase in burnout [61]. Edwards and colleagues examined 715 small-to-medium-size non-VA primary care practices, and found that those with zero burnout had better working environments and used more quality improvement strategies, compared to high burnout practices [62]. These results suggest that interventions that empower and engage providers and staff may be key to improving organizational climate and reducing burnout.

In conclusion, burnout and turnover intent among VA primary care HCWs was high, but lower than pre-pandemic trends during the first six months of the COVID-19 pandemic, and COVID-specific organizational contextual factors were not associated with high burnout or turnover intent. We found that highly engaged providers and staff were less likely to be burned out or to intend to leave their jobs. Those with reasonable workloads, and positive perceptions of their leadership and workgroups were also less likely to be burned out. Interventions to improve organizational climate, and increase job resources and engagement, should be developed and evaluated, with the intent of buffering the impacts of external and organizational contextual factors on burnout. Improving primary care HCW working conditions now may be the key to protecting against high burnout in the face of future challenges, whether they be pandemics or other healthcare crises.

## Data Availability

The data that support the findings of this study are available from the VA Office of Primary Care, but this data is not publicly available outside of the VA. Data are however available for VA employees from the authors upon reasonable request and with permission of the VA Office of Primary Care. Interested parties should contact the corresponding author, Eric Apaydin, PhD, MPP, MS, at eric.apaydin@va.gov with their request.
